# 1-(Naphthalen-1-yl)-3-[(thio­phen-2-yl)carbon­yl]thio­urea

**DOI:** 10.1107/S1600536812035350

**Published:** 2012-09-08

**Authors:** Durga P. Singh, Seema Pratap, Sushil K. Gupta, Ray J. Butcher

**Affiliations:** a Department of Chemistry, M. M. V., Banaras Hindu University, Varanasi 221 005, India; b School of Studies in Chemistry, Jiwaji University, Gwalior 474 011, India; c Department of Chemistry, Howard University, 525 College Street NW, Washington, DC 20059, USA

## Abstract

In the title compound, C_16_H_12_N_2_OS_2_, the dihedral angles between the mean planes of the central thio­urea core and the thio­phene ring and the naphthalene ring system are 1.8 (2) and 6.45 (18)°, respectively. The mol­ecule adopts a *trans–cis* conformation with respect to the position of thio­phenoyl and naphthyl groups relative to the S atom across the thiourea C—N bonds. Both the thio­phene ring and the sulfanyl­idene S atom are disordered over two sets of sites with occupancies of 0.862 (3):0.138 (3) and 0.977 (3):0.023 (3), respectively. An intra­molecular N—H⋯O hydrogen bond is observed. The crystal packing features two N—H⋯S hydrogen bonds.

## Related literature

For heterocyclic thiourea derivatives, metal complexes and their applications, see: D’hooghe *et al.* (2005 ▶); Aly *et al.* (2007 ▶); Estévez-Hernández *et al.* (2007 ▶); Saeed *et al.* (2008*a* ▶,*b* ▶,*c* ▶). For related structures, see: Singh *et al.* (2012 ▶); Koch (2001 ▶); Pérez *et al.* (2008 ▶). For the synthesis, see: Otazo-Sánchez *et al.* (2001 ▶).
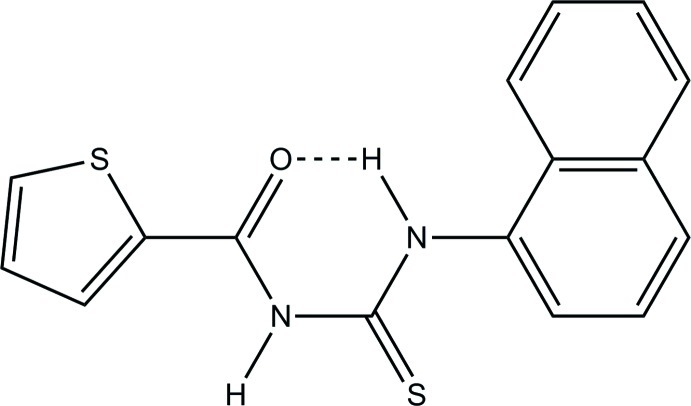


## Experimental

### 

#### Crystal data

C_16_H_12_N_2_OS_2_
*M* *_r_* = 312.40
Monoclinic, 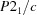

*a* = 14.929 (2) Å
*b* = 5.9086 (8) Å
*c* = 17.071 (3) Å
β = 104.030 (14)°
*V* = 1460.9 (4) Å^3^
*Z* = 4
Mo *K*α radiation
μ = 0.36 mm^−1^
*T* = 173 K
0.35 × 0.25 × 0.15 mm

#### Data collection

Oxford Diffraction Xcalibur Eos diffractometer
Absorption correction: multi-scan (*CrysAlis PRO*; Oxford Diffraction, 2007 ▶) *T*_min_ = 0.712, *T*_max_ = 1.000
4822 measured reflections
2625 independent reflections
1626 reflections with *I* > 2σ(*I*)
*R*_int_ = 0.052

#### Refinement

*R*[*F* ^2^ > 2σ(*F* ^2^)] = 0.049
*wR*(*F* ^2^) = 0.165
*S* = 1.09
2625 reflections
210 parameters
10 restraints
H-atom parameters constrained
Δρ_max_ = 0.33 e Å^−3^
Δρ_min_ = −0.31 e Å^−3^

### 

Data collection: *CrysAlis PRO* (Oxford Diffraction, 2007 ▶); cell refinement: *CrysAlis PRO*; data reduction: *CrysAlis PRO*; program(s) used to solve structure: *SHELXS97* (Sheldrick, 2008 ▶); program(s) used to refine structure: *SHELXL97* (Sheldrick, 2008 ▶); molecular graphics: *SHELXTL* (Sheldrick, 2008 ▶); software used to prepare material for publication: *SHELXTL*.

## Supplementary Material

Crystal structure: contains datablock(s) global, I. DOI: 10.1107/S1600536812035350/bq2371sup1.cif

Structure factors: contains datablock(s) I. DOI: 10.1107/S1600536812035350/bq2371Isup2.hkl

Supplementary material file. DOI: 10.1107/S1600536812035350/bq2371Isup3.cml

Additional supplementary materials:  crystallographic information; 3D view; checkCIF report

## Figures and Tables

**Table 1:** Hydrogen-bond geometry (Å, °)

*D*—H⋯*A*	*D*—H	H⋯*A*	*D*⋯*A*	*D*—H⋯*A*
N1—H1*A*⋯O1	0.88	1.86	2.615 (3)	143
N2—H2*A*⋯S2*B*	0.88	2.57	3.026 (14)	113
N2—H2*A*⋯S1*A* ^i^	0.88	2.56	3.41 (4)	164
N2—H2*A*⋯S1*B* ^i^	0.88	2.80	3.557 (3)	145

Symmetry code: (i) 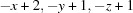.

**Fig. 1.**
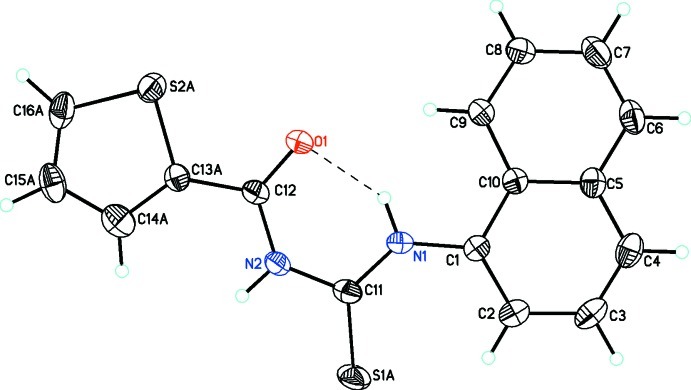
Molecular structure of the title compound (major component only) showing the atom labeling scheme and 30% probability displacement ellipsoids. Dashed lines indicate an intramolecular N—H···O hydrogen bond.

**Fig. 2.**
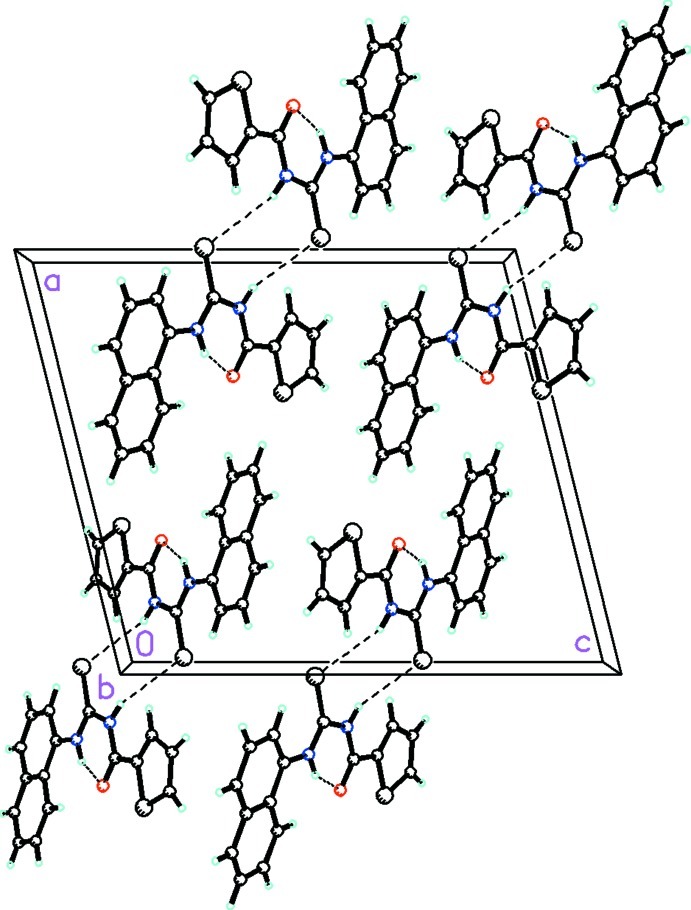
Crystal packing for the title compound viewed along b axis. Dashed lines indicate intramolecular N—H···O and intermolecular N—H···S hydrogen bonds.

## supplementary crystallographic information

### Comment

Aroylythiourea and its derivatives are an important class of organic compounds due to their ability to form a variety of heterocyclic compounds (D'hooghe *et al.*, 2005) and metal complexes that can be used as ionophores in potentiometric and amperometric sensors (Aly *et al.*, 2007; Estévez-Hernández *et al.*, 2007) and as epoxy resin curing agents and accelerators (Saeed *et al.*, 2008*a*, 2008*b*, 2008*c*). The title compound, *N*-(naphthalen-1-yl)-3-oxo-3-(thiophen-2-yl)propanethioamide is an important precursor with O and S as potential donor sites, and can be used to form heterocycles and metal complexes. We have reported recently the synthesis and crystal structure of methyl 2-(thiophene-2-carboxamido)benzoate (Singh *et al.*, 2012). We herein report the synthesis and crystal structure of the biologically active title compound.

In the title compound (Fig.1) the bond lengths and angles are within the ranges observed for similar compounds (Koch, 2001; Pérez *et al.*, 2008). The C11—S1B [1.661 (3) Å] and C12—O1 [1.230 (3) Å] bonds show typical double-bond character. However, the C—N bond lengths, C12—N2 [1.376 (4) Å], C11—N1 [1.326 (4) Å], C11—N2 [1.397 (4) Å] and C1—N1 [1.419 (4) Å] are all shorter than the normal C—N single-bond length of about 1.48 Å and indicate some degree of delocalization. The central thiourea fragment (N1/C11/N2/C12/O1) makes a dihedral angle of 2.03 (41)° with the major part of the 2-thiophenoyl group (S2/C13/C14/C15/C16) and 6.51 (16)° with the naphthalene ring (C1/C2/C3/C4/C5/C6/C7/C8/C9/C10), respectively. Thus, the conformation is almost planar and adopts a *trans-cis* configuration with respect to the position of the thiophenoyl and naphthyl groups relative to the S atom across the thiourea C—N bonds. This geometry is stabilized by both an N1—H1···O1 intramolecular hydrogen bond and two intermolecular N—H···S hydrogen bonds (Fig.2). In addition, both the thiophene ring and the thio S are disordered over two positions with occupancies of 0.862 (3)/0.138 (3) and 0.977 (3)/0.023 (3), respectively.

### Experimental

The title compound was synthesized according to a previous report (Otazo-Sánchez *et al.*, 2001), by converting furoyl choride into furoyl isothiocyanate and then condensing with α-naphthylamine. The resulting solid product was crystallized from ethanol yielding X-ray quality single crystals (M.P.: 459 K). Anal. Calc. for C_16_H_12_N_2_OS_2_ (%): C, 61.51; H, 3.87; N, 8.97. Found: C, 61.20; H, 3.80; N, 9.10.

### Refinement

H1 was located by a Fourier map and refined isotropically. All of the remaining H atoms were placed in their calculated positions and then refined using the riding model with Atom—H lengths of 0.93 Å (CH). Isotropic displacement parameters for these atoms were set to 1.20 (CH) times *U*_eq_ of the parent atom. Both the thiophene ring and the thio S are disordered over two positions with occupancies of 0.867 (3)/0.133 (3) and 0.84 (3)/0.16 (3), respectively.

### Crystal data

**Table:**

C_16_H_12_N_2_OS_2_	*F*(000) = 648
*M**_r_* = 312.40	*D*_x_ = 1.420 Mg m^−^^3^
Monoclinic, *P*2_1_/*c*	Mo *K*α radiation, λ = 0.71073 Å
*a* = 14.929 (2) Å	Cell parameters from 1174 reflections
*b* = 5.9086 (8) Å	θ = 3.3–27.3°
*c* = 17.071 (3) Å	µ = 0.36 mm^−^^1^
β = 104.030 (14)°	*T* = 173 K
*V* = 1460.9 (4) Å^3^	Prism, colorless
*Z* = 4	0.35 × 0.25 × 0.15 mm

### Data collection

**Table:**

Oxford Diffraction Xcalibur Eos diffractometer	2625 independent reflections
Radiation source: Enhance (Mo) X-ray Source	1626 reflections with *I* > 2σ(*I*)
Graphite monochromator	*R*_int_ = 0.052
Detector resolution: 16.0938 pixels mm^-1^	θ_max_ = 25.5°, θ_min_ = 3.7°
ω scans	*h* = −18→10
Absorption correction: multi-scan (*CrysAlis PRO*; Oxford Diffraction, 2007)	*k* = −6→7
*T*_min_ = 0.712, *T*_max_ = 1.000	*l* = −17→20
4822 measured reflections	

### Refinement

**Table:**

Refinement on *F*^2^	Primary atom site location: structure-invariant direct methods
Least-squares matrix: full	Secondary atom site location: difference Fourier map
*R*[*F*^2^ > 2σ(*F*^2^)] = 0.049	Hydrogen site location: inferred from neighbouring sites
*wR*(*F*^2^) = 0.165	H-atom parameters constrained
*S* = 1.09	*w* = 1/[σ^2^(*F*_o_^2^) + (0.058*P*)^2^ + 0.3695*P*] where *P* = (*F*_o_^2^ + 2*F*_c_^2^)/3
2625 reflections	(Δ/σ)_max_ < 0.001
210 parameters	Δρ_max_ = 0.33 e Å^−^^3^
10 restraints	Δρ_min_ = −0.31 e Å^−^^3^

### Special details

**Table:**

Geometry. All esds (except the esd in the dihedral angle between two l.s. planes) are estimated using the full covariance matrix. The cell esds are taken into account individually in the estimation of esds in distances, angles and torsion angles; correlations between esds in cell parameters are only used when they are defined by crystal symmetry. An approximate (isotropic) treatment of cell esds is used for estimating esds involving l.s. planes.
Refinement. Refinement of *F*^2^ against ALL reflections. The weighted *R*-factor *wR* and goodness of fit *S* are based on *F*^2^, conventional *R*-factors *R* are based on *F*, with *F* set to zero for negative *F*^2^. The threshold expression of *F*^2^ > σ(*F*^2^) is used only for calculating *R*-factors(gt) *etc*. and is not relevant to the choice of reflections for refinement. *R*-factors based on *F*^2^ are statistically about twice as large as those based on *F*, and *R*- factors based on ALL data will be even larger.

### Fractional atomic coordinates and isotropic or equivalent isotropic displacement parameters (Å^2^)

**Table:**

	*x*	*y*	*z*	*U*_iso_*/*U*_eq_	Occ. (<1)
S1A	0.962 (3)	0.732 (11)	0.421 (4)	0.0764 (5)	0.023 (3)
S1B	0.98334 (7)	0.6323 (3)	0.38685 (9)	0.0764 (5)	0.977 (3)
S2A	0.66455 (8)	−0.0348 (2)	0.46547 (8)	0.0582 (4)	0.862 (3)
C13A	0.7707 (3)	0.0902 (7)	0.4715 (3)	0.0404 (11)	0.862 (3)
C14A	0.8378 (4)	−0.0413 (13)	0.5210 (5)	0.0655 (19)	0.862 (3)
H14A	0.9018	−0.0083	0.5310	0.079*	0.862 (3)
C15A	0.8041 (4)	−0.2228 (11)	0.5543 (4)	0.0655 (15)	0.862 (3)
H15A	0.8419	−0.3270	0.5900	0.079*	0.862 (3)
C16A	0.7114 (4)	−0.2380 (12)	0.5309 (6)	0.0695 (14)	0.862 (3)
H16A	0.6765	−0.3515	0.5496	0.083*	0.862 (3)
S2B	0.8612 (8)	−0.036 (2)	0.5239 (9)	0.0582 (4)	0.138 (3)
C13B	0.7593 (17)	0.051 (5)	0.4590 (19)	0.0404 (11)	0.138 (3)
C14B	0.6895 (17)	−0.046 (5)	0.488 (2)	0.0655 (19)	0.138 (3)
H14B	0.6294	0.0174	0.4803	0.079*	0.138 (3)
C15B	0.716 (2)	−0.242 (7)	0.530 (4)	0.0655 (15)	0.138 (3)
H15B	0.6767	−0.3658	0.5338	0.079*	0.138 (3)
C16B	0.807 (2)	−0.233 (7)	0.566 (3)	0.0695 (14)	0.138 (3)
H16B	0.8373	−0.3273	0.6097	0.083*	0.138 (3)
O1	0.69721 (14)	0.3590 (3)	0.37691 (14)	0.0546 (6)	
N1	0.79871 (16)	0.6726 (4)	0.33310 (16)	0.0440 (7)	
H1A	0.7462	0.6094	0.3360	0.053*	
N2	0.85320 (16)	0.3884 (4)	0.42315 (16)	0.0452 (7)	
H2A	0.9009	0.3300	0.4579	0.054*	
C1	0.7879 (2)	0.8627 (5)	0.28085 (19)	0.0432 (8)	
C2	0.8584 (2)	1.0074 (5)	0.2753 (2)	0.0541 (9)	
H2	0.9196	0.9774	0.3054	0.065*	
C3	0.8401 (3)	1.1985 (5)	0.2255 (2)	0.0585 (10)	
H3	0.8894	1.2973	0.2223	0.070*	
C4	0.7537 (3)	1.2454 (5)	0.1817 (2)	0.0570 (9)	
H4	0.7429	1.3778	0.1492	0.068*	
C5	0.6798 (2)	1.0997 (5)	0.1838 (2)	0.0468 (8)	
C6	0.5901 (3)	1.1427 (6)	0.1368 (2)	0.0594 (10)	
H6	0.5788	1.2741	0.1038	0.071*	
C7	0.5189 (3)	0.9981 (6)	0.1379 (2)	0.0659 (11)	
H7	0.4587	1.0297	0.1060	0.079*	
C8	0.5347 (2)	0.8048 (6)	0.1857 (2)	0.0574 (10)	
H8	0.4851	0.7044	0.1859	0.069*	
C9	0.6200 (2)	0.7578 (5)	0.2319 (2)	0.0494 (9)	
H9	0.6291	0.6249	0.2642	0.059*	
C10	0.6962 (2)	0.9031 (5)	0.23326 (19)	0.0416 (8)	
C11	0.8727 (2)	0.5719 (5)	0.37841 (19)	0.0434 (8)	
C12	0.7696 (2)	0.2864 (5)	0.4203 (2)	0.0433 (8)	

### Atomic displacement parameters (Å^2^)

**Table:**

	*U*^11^	*U*^22^	*U*^33^	*U*^12^	*U*^13^	*U*^23^
S1A	0.0282 (5)	0.1166 (10)	0.0801 (9)	−0.0049 (6)	0.0048 (5)	0.0291 (8)
S1B	0.0282 (5)	0.1166 (10)	0.0801 (9)	−0.0049 (6)	0.0048 (5)	0.0291 (8)
S2A	0.0543 (7)	0.0566 (6)	0.0598 (9)	−0.0107 (6)	0.0061 (5)	0.0071 (6)
C13A	0.0405 (19)	0.035 (2)	0.041 (2)	−0.0003 (16)	0.0015 (16)	−0.0068 (18)
C14A	0.057 (4)	0.064 (3)	0.077 (4)	0.000 (3)	0.020 (4)	−0.010 (2)
C15A	0.091 (4)	0.048 (2)	0.051 (3)	0.023 (3)	0.004 (3)	0.006 (2)
C16A	0.090 (4)	0.053 (3)	0.063 (3)	−0.015 (3)	0.015 (3)	0.002 (2)
S2B	0.0543 (7)	0.0566 (6)	0.0598 (9)	−0.0107 (6)	0.0061 (5)	0.0071 (6)
C13B	0.0405 (19)	0.035 (2)	0.041 (2)	−0.0003 (16)	0.0015 (16)	−0.0068 (18)
C14B	0.057 (4)	0.064 (3)	0.077 (4)	0.000 (3)	0.020 (4)	−0.010 (2)
C15B	0.091 (4)	0.048 (2)	0.051 (3)	0.023 (3)	0.004 (3)	0.006 (2)
C16B	0.090 (4)	0.053 (3)	0.063 (3)	−0.015 (3)	0.015 (3)	0.002 (2)
O1	0.0340 (12)	0.0537 (13)	0.0694 (16)	−0.0034 (10)	−0.0006 (11)	0.0150 (12)
N1	0.0286 (13)	0.0453 (14)	0.0541 (17)	−0.0030 (11)	0.0021 (12)	0.0007 (13)
N2	0.0302 (13)	0.0547 (15)	0.0472 (16)	0.0030 (12)	0.0026 (11)	0.0113 (13)
C1	0.0381 (17)	0.0383 (15)	0.0503 (19)	−0.0019 (14)	0.0050 (14)	−0.0047 (14)
C2	0.0455 (19)	0.0503 (18)	0.067 (2)	−0.0095 (16)	0.0136 (17)	−0.0035 (17)
C3	0.063 (2)	0.0504 (18)	0.068 (2)	−0.0188 (18)	0.0281 (19)	−0.0075 (18)
C4	0.072 (2)	0.0452 (17)	0.059 (2)	−0.0030 (19)	0.0254 (19)	0.0032 (16)
C5	0.0516 (19)	0.0406 (16)	0.050 (2)	0.0058 (16)	0.0165 (16)	−0.0023 (15)
C6	0.062 (2)	0.057 (2)	0.059 (2)	0.0175 (19)	0.0146 (19)	0.0156 (18)
C7	0.051 (2)	0.075 (2)	0.067 (3)	0.018 (2)	0.0062 (19)	0.017 (2)
C8	0.0409 (19)	0.064 (2)	0.065 (2)	0.0007 (17)	0.0078 (17)	0.0111 (19)
C9	0.0421 (18)	0.0506 (17)	0.052 (2)	−0.0010 (16)	0.0044 (15)	0.0080 (16)
C10	0.0387 (17)	0.0380 (15)	0.0477 (19)	0.0024 (14)	0.0092 (14)	−0.0061 (14)
C11	0.0342 (16)	0.0593 (18)	0.0356 (17)	−0.0004 (15)	0.0065 (13)	0.0002 (15)
C12	0.0322 (16)	0.0423 (16)	0.0513 (19)	0.0028 (14)	0.0023 (14)	−0.0017 (15)

### Geometric parameters (Å, º)

**Table:**

S1A—C11	1.65 (5)	N1—H1A	0.8800
S1B—C11	1.661 (3)	N2—C12	1.376 (4)
S2A—C16A	1.674 (6)	N2—C11	1.397 (4)
S2A—C13A	1.729 (4)	N2—H2A	0.8800
C13A—C14A	1.383 (7)	C1—C2	1.377 (4)
C13A—C12	1.449 (5)	C1—C10	1.432 (4)
C14A—C15A	1.365 (8)	C2—C3	1.401 (5)
C14A—H14A	0.9500	C2—H2	0.9500
C15A—C16A	1.347 (6)	C3—C4	1.353 (5)
C15A—H15A	0.9500	C3—H3	0.9500
C16A—H16A	0.9500	C4—C5	1.408 (5)
S2B—C16B	1.67 (2)	C4—H4	0.9500
S2B—C13B	1.728 (19)	C5—C6	1.407 (4)
C13B—C14B	1.39 (2)	C5—C10	1.422 (4)
C13B—C12	1.56 (3)	C6—C7	1.367 (5)
C14B—C15B	1.37 (2)	C6—H6	0.9500
C14B—H14B	0.9500	C7—C8	1.390 (5)
C15B—C16B	1.350 (18)	C7—H7	0.9500
C15B—H15B	0.9500	C8—C9	1.354 (4)
C16B—H16B	0.9500	C8—H8	0.9500
O1—C12	1.230 (3)	C9—C10	1.421 (4)
N1—C11	1.326 (4)	C9—H9	0.9500
N1—C1	1.419 (4)		
			
C16A—S2A—C13A	92.2 (2)	C3—C2—H2	119.9
C14A—C13A—C12	136.0 (5)	C4—C3—C2	121.3 (3)
C14A—C13A—S2A	108.3 (4)	C4—C3—H3	119.3
C12—C13A—S2A	115.4 (3)	C2—C3—H3	119.3
C15A—C14A—C13A	114.2 (5)	C3—C4—C5	120.6 (3)
C15A—C14A—H14A	122.9	C3—C4—H4	119.7
C13A—C14A—H14A	122.9	C5—C4—H4	119.7
C16A—C15A—C14A	112.6 (5)	C4—C5—C6	121.3 (3)
C16A—C15A—H15A	123.7	C4—C5—C10	119.4 (3)
C14A—C15A—H15A	123.7	C6—C5—C10	119.3 (3)
C15A—C16A—S2A	112.5 (5)	C7—C6—C5	121.0 (3)
C15A—C16A—H16A	123.7	C7—C6—H6	119.5
S2A—C16A—H16A	123.7	C5—C6—H6	119.5
C16B—S2B—C13B	92.5 (12)	C6—C7—C8	119.9 (3)
C14B—C13B—C12	133 (2)	C6—C7—H7	120.0
C14B—C13B—S2B	105.6 (15)	C8—C7—H7	120.0
C12—C13B—S2B	112.0 (16)	C9—C8—C7	120.8 (3)
C15B—C14B—C13B	113 (2)	C9—C8—H8	119.6
C15B—C14B—H14B	123.6	C7—C8—H8	119.6
C13B—C14B—H14B	123.6	C8—C9—C10	121.4 (3)
C16B—C15B—C14B	110 (2)	C8—C9—H9	119.3
C16B—C15B—H15B	125.2	C10—C9—H9	119.3
C14B—C15B—H15B	125.2	C9—C10—C5	117.5 (3)
C15B—C16B—S2B	112 (2)	C9—C10—C1	124.0 (3)
C15B—C16B—H16B	124.2	C5—C10—C1	118.5 (3)
S2B—C16B—H16B	124.2	N1—C11—N2	114.4 (3)
C11—N1—C1	132.4 (3)	N1—C11—S1A	118 (2)
C11—N1—H1A	113.8	N2—C11—S1A	117.1 (18)
C1—N1—H1A	113.8	N1—C11—S1B	128.6 (3)
C12—N2—C11	128.9 (2)	N2—C11—S1B	117.0 (2)
C12—N2—H2A	115.5	S1A—C11—S1B	33 (3)
C11—N2—H2A	115.5	O1—C12—N2	121.7 (3)
C2—C1—N1	124.2 (3)	O1—C12—C13A	121.5 (3)
C2—C1—C10	119.9 (3)	N2—C12—C13A	116.8 (3)
N1—C1—C10	115.9 (3)	O1—C12—C13B	113.8 (9)
C1—C2—C3	120.2 (3)	N2—C12—C13B	123.8 (9)
C1—C2—H2	119.9	C13A—C12—C13B	11.9 (13)
			
C16A—S2A—C13A—C14A	3.3 (6)	C4—C5—C10—C9	−178.4 (3)
C16A—S2A—C13A—C12	178.1 (5)	C6—C5—C10—C9	0.4 (5)
C12—C13A—C14A—C15A	−176.1 (6)	C4—C5—C10—C1	1.4 (4)
S2A—C13A—C14A—C15A	−2.9 (8)	C6—C5—C10—C1	−179.8 (3)
C13A—C14A—C15A—C16A	0.8 (11)	C2—C1—C10—C9	176.9 (3)
C14A—C15A—C16A—S2A	1.9 (11)	N1—C1—C10—C9	−4.5 (5)
C13A—S2A—C16A—C15A	−3.0 (8)	C2—C1—C10—C5	−2.8 (5)
C16B—S2B—C13B—C14B	13 (4)	N1—C1—C10—C5	175.8 (3)
C16B—S2B—C13B—C12	164 (3)	C1—N1—C11—N2	178.7 (3)
C12—C13B—C14B—C15B	−169 (4)	C1—N1—C11—S1A	35 (3)
S2B—C13B—C14B—C15B	−27 (5)	C1—N1—C11—S1B	−2.6 (5)
C13B—C14B—C15B—C16B	30 (6)	C12—N2—C11—N1	7.1 (5)
C14B—C15B—C16B—S2B	−19 (7)	C12—N2—C11—S1A	151 (3)
C13B—S2B—C16B—C15B	3 (5)	C12—N2—C11—S1B	−171.6 (3)
C11—N1—C1—C2	−9.6 (5)	C11—N2—C12—O1	−3.4 (5)
C11—N1—C1—C10	171.8 (3)	C11—N2—C12—C13A	177.1 (3)
N1—C1—C2—C3	−176.2 (3)	C11—N2—C12—C13B	165.9 (17)
C10—C1—C2—C3	2.3 (5)	C14A—C13A—C12—O1	174.0 (6)
C1—C2—C3—C4	−0.2 (5)	S2A—C13A—C12—O1	1.2 (5)
C2—C3—C4—C5	−1.3 (5)	C14A—C13A—C12—N2	−6.5 (8)
C3—C4—C5—C6	−178.1 (3)	S2A—C13A—C12—N2	−179.4 (3)
C3—C4—C5—C10	0.7 (5)	C14A—C13A—C12—C13B	122 (7)
C4—C5—C6—C7	178.6 (4)	S2A—C13A—C12—C13B	−51 (6)
C10—C5—C6—C7	−0.2 (5)	C14B—C13B—C12—O1	−36 (4)
C5—C6—C7—C8	−0.3 (6)	S2B—C13B—C12—O1	−176.6 (15)
C6—C7—C8—C9	0.4 (6)	C14B—C13B—C12—N2	154 (3)
C7—C8—C9—C10	−0.2 (5)	S2B—C13B—C12—N2	13 (3)
C8—C9—C10—C5	−0.3 (5)	C14B—C13B—C12—C13A	96 (9)
C8—C9—C10—C1	180.0 (3)	S2B—C13B—C12—C13A	−44 (5)

### Hydrogen-bond geometry (Å, º)

**Table:**

*D*—H···*A*	*D*—H	H···*A*	*D*···*A*	*D*—H···*A*
N1—H1*A*···O1	0.88	1.86	2.615 (3)	143
N2—H2*A*···S2*B*	0.88	2.57	3.026 (14)	113
N2—H2*A*···S1*A*^i^	0.88	2.56	3.41 (4)	164
N2—H2*A*···S1*B*^i^	0.88	2.80	3.557 (3)	145

Symmetry code: (i) −*x*+2, −*y*+1, −*z*+1.
